# Herbal Active Ingredients: An Emerging Potential for the Prevention and Treatment of Papillary Thyroid Carcinoma

**DOI:** 10.1155/2020/1340153

**Published:** 2020-01-31

**Authors:** Yang Yang, Qin Chen, Wen-Ying Yu, Huan-Huan Zhang, Yu-Sen Zhong, Song-Zhao Zhang, Jia-Feng Wang, Chen-Huan Yu

**Affiliations:** ^1^Key Laboratory of Experimental Animal and Safety Evaluation, Zhejiang Academy of Medical Sciences, Hangzhou 310013, China; ^2^Department of Clinical Laboratory Medicine, The Second Affiliated Hospital of Medicine School, Zhejiang University, Hangzhou 310009, China

## Abstract

Papillary thyroid carcinoma (PTC) is the most common subtype of differentiated thyroid cancers in Asian coastal cities, where the patients have increased risk of potentially high or excessive iodine intake. Given the high metastasis and recurrence of patients with BRAF^V600E^ mutation, the mortality rate of thyroid cancer has recently shown an upward trend. A variety of therapies, including surgery, radiotherapy, and chemotherapy, have been used to treat thyroid cancer, but these therapies still have limitations, including postoperative complications, drug resistance, poor efficacy, or serious side effects. Recent studies have shown the potential of active ingredients derived from herbal medicine in inhibiting PTC via various cell signaling pathways. Some plant-derived compounds, such as apigenin, genistein, and curcumin, are also known to prevent and treat PTC. This article summarizes the recent advances in the structure-functional impact of anti-PTC active ingredients and their effects on PTC cells and tumor microenvironments with an emphasis on their challenges from basic research to clinical practice.

## 1. Introduction

Thyroid cancer is the most common endocrine neoplasms accounting for 5.0% of head and neck cancers [1]. Studies have shown that approximately 95% of thyroid cancers originate from thyroid follicular epithelial cells, including papillary thyroid carcinoma (PTC), follicular thyroid carcinoma (FTC), and anaplastic thyroid cancer (ATC); in addition, a small amount is medullary thyroid carcinoma (MTC) originated from parafollicular cells in the thyroid gland [2, 3]. Among those subtypes, approximately 70% to 80% of all types of thyroid cancers is PTC. Epidemiological studies have revealed that PTC prevalence has increased at an average annual rate of nearly 4% in recent years, and most patients with thyroid cancer suffer from PTC, which is also the main driver of the increased incidence of thyroid cancer [4–7]. PTC has become one of the seven major causes of new malignant tumors among women, and almost all thyroid cancers among children are classified as PTC [8, 9]. Among patients with PTC, the accompanying cervical lymph node metastasis rate reaches 5.4% to 13% after surgery [10–12]. The most common clinical therapies being used for managing PTC include surgery, chemotherapy, and physiotherapy, all of which are hindered by recurrence and metastasis.

With irregular living habits (such as sleep deprivation and long-term high calorie diet intaking) and environmental factors (such as electromagnetic radiation), endocrine disorders including thyroid dysfunction become more and more common to human beings [13, 14]. Both genetic and environmental factors act on thyroid cells and ultimately lead to the transformation of normal thyroid cells into tumor cells. During PTC pathogenesis, some critical genes (including BRAF, RET, KRAS, and PI3KCA) through mutation or chromosomal translocation continuously activate their dependent downstream signaling pathways, such as mitogen-activated protein kinase (MAPK), phosphoinositide 3-kinase (PI3K)/AKT, nuclear factor-*κ*B (NF-*κ*B), and Notch-1, and thereby lead to cellular proliferation, migration, invasion, and angiogenesis [15–17]. Recently, emerging clinical trials and experimental researches also demonstrated that some noncoding RNA expressions, such as miRs-21, -34b, -221/222, lncRNA ATB, lncRNA H19, lncRNA HOXA-AS2, circITCH, and circZFR, showed significant association with aggressive clinicopathologic feature in PTC, including tumor size, lymphovascular invasion, lymph node metastases, and presence of BRAF^V600E^ mutation [17–21]. Despite the advances in tumorigenesis, metastasis, and therapy, the underlying mechanism of PTC remains unclear. Therefore, further studies on the pathogenesis, prevention, and treatment of PTC in the pharmaceutical circle should be conducted.

Traditional herbal medicine has an important position in PTC prevention and treatment in Asian countries for a long history. Many active ingredients derived from food and herbs could prevent the development of PTC. Characterized by their mildness and long-lasting action, limited side effects, long-term use, and multitarget regulation, these herbal active ingredients provide many advantages and cannot be replaced by western medicine [22–24]. They not only inhibit the proliferation and promote the apoptosis of PTC cells by regulating critical signal pathways but also improve the immunity of patients and decrease stress response [25–27] as shown in Table 1. This study discusses the role of phytochemicals in thyroid signaling modulation and their possible beneficial or unfavorable effects on the risk of thyroid cancer.

## 2. Tannins (Phenolic Acids)

On the basis of their chemical structure, tannins can be categorized into hydrolyzable, condensed, and complex tannins. Condensed tannins manifest numerous pharmacological effects, such as antioxidant, antitumor, antihuman immunodeficiency virus, anti-inflammatory, and antimicrobial properties, and are widely found in many medicinal plants and dietary sources, including fruits, nuts, grains, spices, and beverages [58, 59]. Similarly, hydrolyzable tannins have a variety of pharmacological effects, such as antiviral, antibacterial, antitumor, hypolipidemic, and antioxidant properties, and serve as pharmacodynamic bases of many commonly used medicinal plants [60–64].

Epigallocatechin-3-gallate (EGCG), which is the major catechin in tea, shows remarkable protective effects against several chronic inflammatory and cardiovascular diseases, such as cancer, obesity, diabetes, myocardial ischemia, bronchitis, and asthma [65–70]. EGCG exerts chemopreventive effects on various tumors and selectively inhibits various cancer cell proliferation, metastasis, and invasion via regulating VEGF, MAPK, PI3K, and Wnt pathways [71–73]. Wu et al. treated human PTC cell lines (TT and TPC-1) and the ATC cell line (ARO) with EGCG at concentrations of 10∼200 *μ*M and observed that EGCG concentration-dependently inhibited the proliferation of these PTC cells and made the cell cycle arrest at the S phase. EGCG also induces the apoptosis of both PTC and ATC cells by inhibiting the EGFR-dependent ERK pathway. In addition, it could inhibit growth and angiogenesis but induce the apoptosis of PTC xenograft tumors in nude mice [28]. De Amicis et al. demonstrated that treatment with EGCG at the doses of 10–60 mM inhibited the proliferation of PTC (FB-2) and FTC (WRO) cell lines through suppressing the phosphorylation of AKT and ERK1/2; furthermore, EGCG reduced cell motility and migration by modulating cell adhesion, reorganizing the actin cytoskeleton, increasing E-cadherin expression, and suppressing SNAIL, ZEB, TWIST, Vimentin, N-cadherin, and *α*5-integrin, thereby indicating that EGCG inhibited the proliferation and epithelial-to-mesenchymal transition (EMT) of PTC cells [29].

Resveratrol is a polyphenolic phytoalexin with antioxidant and chemopreventive activities [74]. This material has a wide spectrum of targets, including COX2, Sirt1, p53, and miR-17/miR-20b [75], and can inhibit multiple cellular signaling pathways, which were associated with carcinogenesis and progression [76]. Plenty of studies over the past decades have shown that resveratrol downregulated thyroid cancer stem cell markers (including aldehyde dehydrogenases (ALDH), SOX2, OCT4, and NANOG), decreased proliferation and invasiveness, induced apoptosis, reduced ALDH-associated cancer cell stemness, and upregulated thyroid differentiation markers TTF-1 and NIS, which contributed to radioiodine uptake in the treatment of aggressive thyroid cancers [30]. Notably, it was more effective on the redifferentiation of PTC than that of ATC with a high CSC content [22, 30].

Punicalagin is a large polyphenol compound extracted from pomegranates and is classified as an ellagitannin, a family of hydrolyzable tannins [77]. Punicalagin not only induces the cell death of the PTC cell line BCPAP by triggering ATM-mediated DNA damage response [31] but also leads to the G0/G1 phase arrest and senescence-associated secretory phenotype by triggering NF-*κ*B activation [32].

Curcumin is a natural polyphenol extracted from *Rhizoma curcumae longae*, which is the main component of *Curcuma longa*. Curcumin is one of the best-selling natural edible pigments all over the world and is widely used as a food additive approved by the World Health Organization and most countries. It had various chemopreventive properties, such as antioxidant, antitumoral, antiviral, anti-inflammatory, antihepatotoxic, antidiabetic, hypolipidemic, and neuroprotective properties [78–82]. Several studies have also revealed that curcumin induced PTC cell BCPAP apoptosis and cell arrested at the G2/M phase with the concentration increased involving in multitargeting mechanisms, including the activation of reactive oxygen species (ROS)-independent DNA damage by recruiting ATM-mediated Chk2-Cdc25C-Cdc2 pathway [33], the activation of endoplasmic reticulum (ER) stress by disrupting intracellular calcium homeostasis [34], the inhibition of the *β*-catenin pathway [35], and the modulation of the mitochondrial Bcl-2/Bax pathway [36]. Furthermore, curcumin inhibits invasion and metastasis in PTC cells by upregulating E-cadherin expression and downregulating matrix metalloproteinase-9 (MMP-9) [37]. Curcumin can also inhibit TGF-*β*1-induced EMT via the downregulating phosphorylation of Smad2/3, which in turn inhibits the metastasis of human PTC BCPAP cells [38]. Hypoxia plays a crucial role in tumor metastasis, which is the leading cause of death in patients with PTC [83]. Curcumin significantly reduces the production of hypoxia-induced ROS and the binding capacity of HIF-1*α* to its downstream oncogenes and weakens the migration of PTC cells under hypoxic conditions [84]. Meanwhile, when combined with sorafenib, curcumin significantly inhibits the apoptosis of FTC133 cells via PI3K/AKT and ERK pathways; moreover, compared with chemotherapy drugs, curcumin has lower cytotoxic effects on normal cells [85]. When cotreated with other natural extracts such as spirulina and Boswellia, curcumin can effectively reduce the size of benign thyroid nodules and restore thyroid hormonal dysfunction, thereby preventing the progress of PTC canceration [86].

## 3. Flavonoids

Flavonoids are a group of phenolic antioxidants with strong biological activity that have been widely used in pharmaceutical and food additives. Some flavonoids, such as soy genistein, naringenin, phloretin, and chrysin, are structurally similar to estrogen and have little or weak estrogen-like effects [87]. These phytoestrogens can affect not only thyroid hormone synthesis but also thyroid hormone metabolism [88–90]. Therefore, the beneficial or adverse effects of flavonoids depend on their target tissue and their daily consumption. However, an excessive intake of phytoestrogens, especially soy isoflavones, can undo any benefits of flavonoids and interfere in the iodination of human thyroid hormones [91, 92].

Many studies have shown that the estrogenic potencies of these compounds depend mainly on the presence/absence of bicyclic and hydroxyl structures. (1) The B ring position of flavonoids affects their estrogen-like activity, and the strongest activity is present on the 3 position; (2) the hydroxyl groups on the 5 position of the A ring increase the activity; (3) the hydroxyl groups on the 5′ position of the B ring reduce the activity; (4) the conjugated double bond on the 2 and 3 positions of the C ring greatly enhances the activity; and (5) both glycosyl and isopropenyl inhibit the activity. Moreover, different flavonoids perform selective functions during the estrogen receptor subtype stimulation [87, 93].

Apigenin and quercetin are flavonoids that are most commonly found in a variety of fruits, vegetables, and herbs [94, 95]. Treatment with apigenin at concentrations of 12.5∼100 *μ*M can inhibit the proliferation of BCPAP cells arrested in the G2/M phase and induce autophagy via ROS-mediated DNA damage [39]. Moreover, combining with apigenin and AKT inhibitors enhances the antitumor effects of radioiodine in both BRAF^V600E^-expressed rat thyroid cells and primary cultured PTC cells from TR*β*^PV/PV^ mice [40]. Unlike those of apigenin, the effects of quercetin on thyroid cells have been disputed. Some studies showed that 1.25∼20 *μ*M of quercetin inhibited normal thyroid cell growth in association with the inhibition of the insulin-induced PI3K/AKT pathway. Moreover, quercetin decreases the expression of thyroid-stimulating-hormone-modulated thyroid-restricted gene sodium/iodide symporter (NIS) [96, 97]. By contrast, treatment with 50∼75 *μ*M quercetin shows an excellent anticancer activity by inducing S phase arrest and apoptosis via Hsp90 and Caspase-3/PARP pathways in BCPAP cells [41, 42]. Similarly, myricetin, of which the B ring presents one more hydroxyl group at the 5′ position compared with quercetin, dependently induces apoptosis and DNA condensation of SNU-790 PTC cells, which also involves caspase-dependent mitochondrial dysfunction [43].

Icariin is the main active ingredient of *Epimedium davidii* Franch. and has gained much attention because of its erectogenic and neurotrophic effects [98]. Recently, many studies have demonstrated the application of icariin on hormone-dependent neoplasia and in the treatment of prostatic, ovarian, and thyroid cancers [44, 99–101]. Icariin can inhibit cell proliferation, migration, and invasion via downregulating miR-625-3p and suppressing PI3K/AKT and ERK pathways in both SW579 and TPC1 cells [39].

Flavokawain B is a hepatotoxic constituent extracted from kava root [102] and shows potent cytotoxicity by inducing ROS-mediated apoptotic and autophagic cell death in various tumor cells [103, 104]. This material also inhibits cell viability, migration, and invasion and causes autophagy via the activation of the AMPK/mTOR pathway in thyroid cancer ARO, WRO, and TPC-1 cells [45].

Genistein is the main active ingredient of *Leguminosae*. This isoflavone inhibits the invasion and metastasis of the PTC-derived BHP10-3 cell (with RET/PTC 1 rearrangement), BCPAP, and IHH4 (with BRAF^V600E^ mutation) by inhibiting *β*-catenin and EMT [46]. However, genistein upregulates most thyroid transcript signals, except for thyroid peroxidase, in zebrafish embryos, thereby indicating potential disruptions [105].

Silibinin is a natural hepatoprotective drug and has excellent antioxidant and anticancer properties. It also induces apoptosis, autophagy, makes cell cycle arrest, and inhibits onco-miRNAs which is involved in the PTC tumorigenesis [106]. Previous studies showed that it suppressed cell migration and MMP-9 expression by regulating the ERK pathway in thyroid cancer cells [47].

## 4. Saponins

Saponins are steroid or triterpenoid glycosides commonly found in plants. Extensive studies have shown that saponins have various pharmacological effects, including hypoglycemic, antitumor, anti-inflammatory, immunomodulatory, and vasoprotective properties, and thus they have been widely used for preventing and treating cardiovascular and immunodeficiency diseases [107]. Ginsenosides are by far the most investigated group of saponins with a triterpenoid dammarane skeleton and are the main active ingredients of the ginseng genus (*Panax ginseng* C. A. Mey. *Panax notoginseng* (Burk.) F. H. Chen and *Panax quinquefolium* L.) in *Araliaceae* and *Gynostemma pentaphyllum* (Thunb) Makino. in *Cucurbitaceae* [108, 109]. At high concentrations (>100 *μ*M), ginsenosides exert cytotoxic and haemolytic effects, while treatment at low concentrations (10∼100 *μ*M) inhibits the proliferation of PTC cells, thereby indicating its multidirectional effects on cancer cells [110]. Despite similarities in the structure of dexamethasone, both 20(S)-protopanaxadiol (PPD)-type ginsenosides (such as Rb1, Rb2, Rc, Rd, Rh2, and Compound K) and 20(S)-protopanaxatriol (PPT)-type ginsenosides (such as Re, Rf, Rg1, and Rh1) do not have any effects on glucocorticoid receptor transactivation or transrepression [111]. However, they exert synergistic anti-inflammatory effects when in combination with glucocorticoids at the low doses [112]. Previous studies show that PPT-type ginsenosides (Rg1 and Rg3) can reduce the proliferation, migration, and invasion of PTC cells by upregulating Cx31 and inhibiting Rho GTPase to an alternate cytoskeleton [48, 49]. Furthermore, Rg3 remarkably reduces the expression of the VEGF-C protein in TPC-1, BCPAP, C643, and Ocut-2c cells and inhibits lymph node metastasis in mice [49]. Structure-activity relationships elucidate the association between chemical structures and the anticancer activities of a series of ginsenosides. As shown in Table 2, the anticancer activities of ginsenosides generally take the descending order of CK > Rg1≈20(S)-Rh2≈20(S)-Rg3 > PPT≈PPD, thereby suggesting that the ginsenoside with less polar chemical structures has stronger cytotoxic effects on PTC cells [50, 51].

## 5. Other Bioactivities

Capsaicin (CAP), a major active component of chili peppers, selectively binds to transient receptor potential vanilloid type 1 (TRPV1). CAP (25–100 *μ*M) dose-dependently suppresses the migration, invasion, and adhesion of BCPAP cells by activating TRPV1 and subsequently regulating EMT [52].

Berberine is an isoquinoline alkaloid mainly isolated from *Coptis chinensis* Franch. and *Berberis wilsonae* Hemsl. et Wils. Numerous studies have shown that it had multiple pharmacological effects, including antimicrobial, hypoglycemic, anti-inflammatory, antifibrotic, and antineoplastic properties. Berberine can also induce mitochondrial apoptosis, trigger G0/G1 cell cycle arrest, and suppress the migration of TPC-1 cells via inhibiting PI3K/AKT and MAPK pathways [113].

Paclitaxel is a tetracyclic diterpenoid derived from the dried roots, branches, leaves, and barks of *Taxus chinensis*. Paclitaxel is a first-line chemotherapeutic agent for PTC patients with a squamous cell carcinoma component, of which response rates are 67% and the clinical benefit rate (PR + SD) is 100%. Therefore, weekly paclitaxel may be applied as effective adjuvant chemotherapy after surgery [114].

Pseudolaric acid B (PAB) is a diterpenoid acid extracted from the root barks or barks near the roots of *Pseudolarix amabilis* (Nelson) Rehd. (*Pinaceae*) and is known as an antitubulin therapeutic agent that can suppress microtubule assembly to inhibit the proliferation of cancer cells [53, 115]. PAB can also inhibit the proliferation, invasion, and migration of SW579 cells by preventing the regulation of Bcl-2 and Beclin-1 expression but decreases the expression of nuclear p53 and induces G_2_/M cell cycle arrest by increasing the ratio of autophagic cells [54].

Shikonin is a natural bioactive naphthoquinone derived from *Lithospermum erythrorhizon* Sieb. et Zucc (also called Zi Cao in Asia). It has been recently considered as a natural food additive and antitumor agent for breast cancer, leukemia, hepatoma, and nonsmall cell lung cancer [55, 56, 116]. Previous studies reveal that shikonin dramatically inhibits the migration and invasion of PTC cells via suppressing EMT and inhibiting the expressions of slug and MMP-2, -9, and -14. Furthermore, shikonin induces PTC cell apoptosis by targeting several signaling pathways, suppressing ERK/AKT and DNMT1, and activating p16/Rb and caspase-3-dependent mitochondrial pathways [57, 117, 118]. The oral administration of 2.0 mg/kg shikonin does not cause any liver injury in mice, thereby indicating its safety [117].

Allicin, as another well-known natural food additive, is mainly isolated from garlic. Allicin inhibits the proliferation of cancer cells, induces cell apoptosis, and increases the intracellular levels of ROS [57, 118]. It can also improve multidrug resistance in thyroid cancer cells via inducing autophagy but inactivating AKT and S6 pathways [119].

## 6. Conclusions and Outlook

Although the problems of overdiagnosis and overtreatment now seem to be acknowledged, how to acquire survival benefit for PTC patients becomes to be a basic challenge [120]. Therefore, it is obviously important to explore new mild strategies to prevent and treat PTC. Phytochemicals have received much attention over the past three decades as potential sources of new candidates for cancer chemoprevention and treatment. Given the fact that many available anticancer drugs are derived from plant substances (e.g., taxol, vinblastine, homoharringtonine, *β*-elemene, indole-3-carbinol) as prototypes, this review focuses on herbal active ingredients with high potentials for the prevention and treatment of PTC. The benefits of these ingredients in PTC prevention and treatment have been well investigated, but their underlying mechanism and direct molecular targets remain unclear. Due to the latent toxicological, low bioavailability, foreseeable multidrug resistance, and deficient clinical trials, the extensive assessment of those untapped natural compounds on humanized immune system mouse models and achievable doses and drug delivery systems compatible for human studies still need to be further explored. Moreover, the development and progression of tumors are strongly associated with the physiological and pathological characteristics of the tumor microenvironment (TME) [121], in which hypoxia, chronic inflammation, oxidative stress, and acidosis contribute to cancer progression, including immune escape, angiogenesis, and metastasis. Given their multihydroxy structures, most phytochemicals with antioxidant and anti-inflammatory potentials can be preponderant on the improvement of TME profiles. According to the different features of cell subsets in TME, the patients could derive benefits from the intervention of multidrug combination. However, the effectiveness of herbal active ingredients on TME has been rarely reported. The application of those strategies might promote the clinical translation of these herbal active ingredients for PTC prevention and treatment.

## Figures and Tables

**Table 1:** Anti-PTC mechanisms of herbal active ingredients.

Phytochemicals	Cell lines/patient	Dose (*μ*M)	Mechanisms	References

EGCG	TPC-1, ARO	10∼200	Induce apoptosis via inhibiting EGFR/RAS/ERK pathway	[28]
	FB-2, WRO	10∼60	Inhibit EMT	[29]

Resveratrol	TPC-1, BCPAP	5∼50	Induce apoptosis and differentiation of CSC	[22, 30]

Punicalagin	BCPAP	12.5∼100	Induce cell death by triggering ATM-mediated DNA damage; inhibit senescent growth via NF-*κ*B pathway.	[31, 32]

Curcumin	TPC-1, BCPAP, K1	12.5–50	Induce apoptosis via (1) induction of ROS-independent DNA damage by triggering an ATM-activated Chk2-Cdc25C-Cdc2 pathway; (2) activation of ER stress by disruption of intracellular calcium homeostasis; (3) inhibition of *β*-catenin pathway; (4) modulation of mitochondrial Bcl-2/Bax pathway.	[33] [34] [35] [36]
	BCPAP	12.5∼50	Inhibit invasion and metastasis via (1) upregulating E-cadherin and downregulating MMP-9; (2) reversing EMT by inhibiting TGF-*β*1/Smad2/3 pathway.	
				[37]
				[38]

Apigenin	BCPAP	12.5∼100	Arrest the cell growth in G_2_/M phase; induce autophagy via ROS-mediated DNA damage.	[39]
	PCCl3 with BRAF^V600E^, primary TPC cells	20	Synergistic effects with akt inhibitor	[40]
Quercetin	BCPAP	50–75	Induce apoptosis via inhibiting Hsp90 and caspase-3/parp pathways	[41, 42]

Myricetin	SNU-790	25∼50	Induce apoptosis via inhibiting the caspase-dependent mitochondrial pathway	[43]

Icariin	SW579, TPC1	20–50	Induce apoptosis via downregulation of miR-625-3p and inactivation of PI3K/Akt and MEK/ERK signaling pathways	[44]

Flavokawain B	ARO, WRO, TPC-1	3.5–25	Induce autophagy via regulating AMPK/mTOR pathway	[45]

Genistein	BHP10-3, BCPAP, IHH4	9.5–300	Inhibit *β*-catenin and EMT	[46]

Silibinin	TPC-1	10–100	Suppress migration and MMP-9 expression via ERK pathway	[47]

Ginsenoside Rg1	IHH-4, BCPAP	5–40	Inhibit cell malignancies by upregulating Cx31	[48]

Ginsenoside Rg3	TPC-1, BCPAP	6.25–100	Inhibit invasion and metastasis via reducing rho GTPase	[49]

Capsaicin	BCPAP	25–100	Inhibit invasion and metastasis via activation of TRPV1 and subsequently regulating EMT	[50]

Berberine	TPC-1	10–160	Induce apoptosis, G_0_/G_1_ cell cycle arrest and migration via PI3K/Akt and MAPK pathways	[51]

Paclitaxel	PTC patient with SCC component	Weekly 80 mg/m^2^	The response rate was 67% and the clinical benefit rate was 100%	[52]

Pseudolaric acid B	SW1579	1.25–5	Induce G_2_/M cell cycle arrest by activating autophagy by decreasing nuclear p53 expression	[53]

Shikonin	8505c, 8305c, FTC133, BCPAP, C643, TPC-1, IHH4, K1, HTori-3	3–6	Induce apoptosis via suppression of ERK/Akt and DNMT1, and activation of p16/Rb and caspase-3-dependent mitochondrial pathways;	[54–56]
			Inhibit migration and invasion by suppressing EMT and expression of slug and MMP-2, -9, and -14.	[54]

Allicin	HTh-7	10	Activating autophagy via inactivation of akt and S6 pathways	[57]

EGCG: Epigallocatechin-3-gallate; ATM: ataxia telangiectasia-mutated; ROS: reactive oxygen species; EMT: epithelial-to-mesenchymal transition; CSC: cancer stem cell; ER: endoplasmic reticulum; PTC: papillary thyroid carcinoma; SCC: squamous cell carcinoma; EGFR: epidermal growth factor receptor.

**Table 2:** The structure-activity relationship of ginsenosides.

Basic structure	Compounds	R1	R2	R3	IC_50_ (*μ*M)^a^
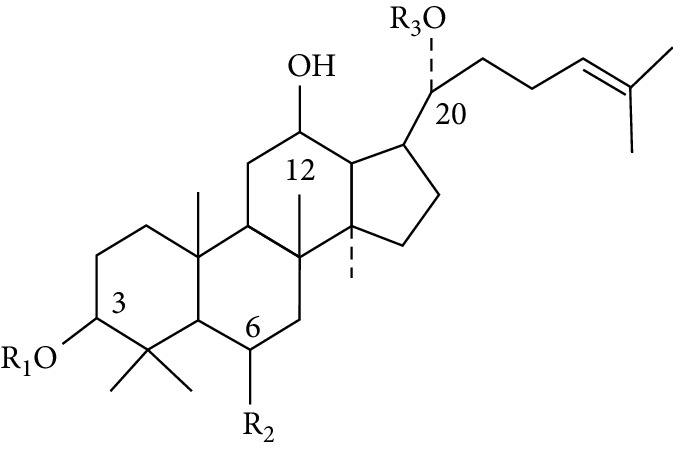	Protopanaxadiol type (PPD)				
	Rb_1_	-Glc^2^-Glc	-H	-Glc^6^-Glc	>200
	Rb_2_	-Glc^2^-Glc	-H	-Glc^6^-Ara (p)	>200
	Rc	-Glc^2^-Glc	-H	-Glc^6^-Ara (f)	>200
	Rd	-Glc^2^-Glc	-H	-Glc	>200
	Rg_3_	-Glc^2^-Glc	-H	-H: 20 (S)	50
	Rh_2_	-Glc	-H	-H: 20 (S)	45
	CK	-Glc	-H	-Glc^6^-Ara (p)	10
	PPD	-H	-H	-H	80
	Protopanaxatriol type (PPT)				
	Re	-Glc^2^-Rha	-OH	-Glc	>200
	Rf	-Glc^2^-Glc	-OH	-H	>200
	Rg_1_	-Glc	-OH	-Glc	40
	Rg_2_ (S)	-Glc^2^-Rha	-OH	-H: 20 (S)	>200
	Rh_1_	-Glc	-OH	-H	>200
	Rg_2_ (R)	-Glc^2^-Rha	-OH	-H: 20 (R)	>200
	PPT	-H	-OH	-H	70

a: inhibition of thyroid cancer cells (for 48 h).
